# A Lost Art in Surgery

**Published:** 1877-07

**Authors:** A. B. Crosby

**Affiliations:** New York


[By A. B. Crosby, M. D., New York.]

A Lost Art in Surgery.—But if this lost art of cleanliness is to be restored, how is it to be accomplished? The moral accountability for the disastrous results likely to follow the want of cleanly precautions in wards, seems to me to rest on the surgeon himself. It is for him, reverently realizing the functions of his high office, to point out the way. To accomplish the desired end, the exact duties in this regard, of surgeons, internes and nurses should be definitely enjoined. The surgeon should then hold his interne to a rigid daily accountability, and he in turn should narrowly watch the nurses. All causes for complaint of neglected duties to be instantly reported to the surgeon, and if not within his province to correct, to be reported by him to the hospital authorities. These details have seemed to me so absolutely essential, that I think they should be printed on cards which should be nailed on every door in the wards, and a copy furnished to every attendant connected with the ward for his instruction and guidance. As, however, the sum of these regulations has been expressed, I shall not weary the Society with them, simply adding that I have divided them into four sections.

*First.*—Regulations to be observed by all persons in common having any official connection with the ward.

*Second.*—Regulations for the guidance of internes.

*Third.*—Regulations for the guidance of nurses.

*Fourth.*—Regulations with reference to general cleanliness, designed for the head of the hospital.

And so finally we have reaffirmed the adage that "cleanliness is next to Godliness," and this, too, in the largest and best sense is *health.—Archives of Clinical Surgery.*

